# Evidence for bystander signalling between human trophoblast cells and human embryonic stem cells

**DOI:** 10.1038/srep11694

**Published:** 2015-07-14

**Authors:** Anna J Jones, Paul J Gokhale, Thomas F Allison, Barry Sampson, Sharan Athwal, Simon Grant, Peter W Andrews, Nicholas D Allen, C Patrick Case

**Affiliations:** 1 Musculoskeletal Research Unit, School of Clinical Sciences (North Bristol), University of Bristol, Bristol BS10 5NB; 2 Centre for Stem Cell Biology, Department of Biomedical Science, The University of Sheffield, Sheffield S10 2TN; 3 GMO 02 Medical Oncology Block, Medical Oncology, Charing Cross Campus, London SW7 2AZ; 4 Department of Obstetrics, Southmead Hospital, Bristol BS10 5NB; 5 Cardiff School of Biosciences, The Sir Martin Evans Building, Museum Avenue, Cardiff, CF10 3AX.

## Abstract

Maternal exposure during pregnancy to toxins can occasionally lead to miscarriage and malformation. It is currently thought that toxins pass through the placental barrier, albeit bi-layered in the first trimester, and damage the fetus directly, albeit at low concentration. Here we examined the responses of human embryonic stem (hES) cells in tissue culture to two metals at low concentration. We compared direct exposures with indirect exposures across a bi-layered model of the placenta cell barrier. Direct exposure caused increased DNA damage without apoptosis or a loss of cell number but with some evidence of altered differentiation. Indirect exposure caused increased DNA damage and apoptosis but without loss of pluripotency. This was not caused by metal ions passing through the barrier. Instead the hES cells responded to signalling molecules (including TNF-α) secreted by the barrier cells. This mechanism was dependent on connexin 43 mediated intercellular ‘bystander signalling’ both within and between the trophoblast barrier and the hES colonies. These results highlight key differences between direct and indirect exposure of hES cells across a trophoblast barrier to metal toxins. It offers a theoretical possibility that an indirectly mediated toxicity of hES cells might have biological relevance to fetal development.

## Significance statement

Exposure to some toxins during pregnancy may increase the risk of miscarriage and fetal malformation. It has been assumed that this is due to a passage of toxin from maternal blood, across the placenta, to directly expose the fetus. Here we show a fundamental difference in the *in vitro* responses of human embryonic stem cells to low doses of toxin according to whether the exposure is direct or indirect, across a bilayered trophoblast barrier in tissue culture. Direct exposure causes DNA damage and cell differentiation without apoptosis. Indirect exposure causes DNA damage and apoptosis without differentiation. This difference is due to bystander signalling both within and between the trophoblast barrier and stem cells. We suggest a theoretical possibility of an additional and novel mechanism for fetal damage.

## Introduction

Occupational or industrial exposure to toxic heavy metals affects millions of humans worldwide^1^,^2^. Exposure of a mother to some of the heavy metals during pregnancy has been linked with adverse effects in the offspring, including genetic damage, trans-generational carcinogenesis, structural abnormalities, resorption of the fetus and miscarriage^1^,^2^,^3^,^4^,^5^,^6^,^7^. The mechanism by which the fetus becomes damaged is unknown. Analyses of umbilical cord blood from exposed mothers have shown that low concentrations of metal are able to cross the placenta. The current view is that these low concentrations may be sufficient to damage the fetus, which is exquisitely sensitive to toxins, especially in critical and early stages of development^8^,^9^,^10^. However, measurement of metal levels in the umbilical cord blood reflects the concentration of metal that is able to cross the placenta at term. The structure of the human placenta changes throughout pregnancy^11^. In the first trimester the placenta barrier is thick, consisting of a layer of syncytiotrophoblast (a syncytium in contact with the maternal blood) that rests on a second layer of mononucleate cytotrophoblast cells. At term it is much thinner and comprised predominantly of a monolayer of syncytiotrophoblast with proportionally much fewer cytotrophoblasts. It also becomes more permeable at term with 7% of the trophoblast surface incomplete^12^. Therefore, the measurement of metal in umbilical cord blood at term may overestimate the exposure of the fetus at an early stage of pregnancy.

In recent years evidence for a metal-induced bystander effect has emerged. Confluent bi-layers of trophoblast cells or corneal epithelial cells, which are exposed to high levels of Co^2+^ and/or Cr^6+^ particles or ions on the apical surface, have been shown to secrete signalling molecules that cause DNA damage in underlying and unexposed fibroblast cells^13^,^14^. Similarly, conditioned medium taken from fibroblast cells or thyroid carcinoma cells, which had been previously exposed to high concentrations of Cr^6+^, induced DNA damage in unexposed fibroblast cells following medium transfer^15^. The exact mechanism for the metal-induced bystander effect is unknown but it has been shown to involve intercellular Ca^2+^ wave propagation, ATP release and the production of cytokines, including IL-6, IL-8 and TNFα^13^,^14^,^15^.

**Figure 1:**
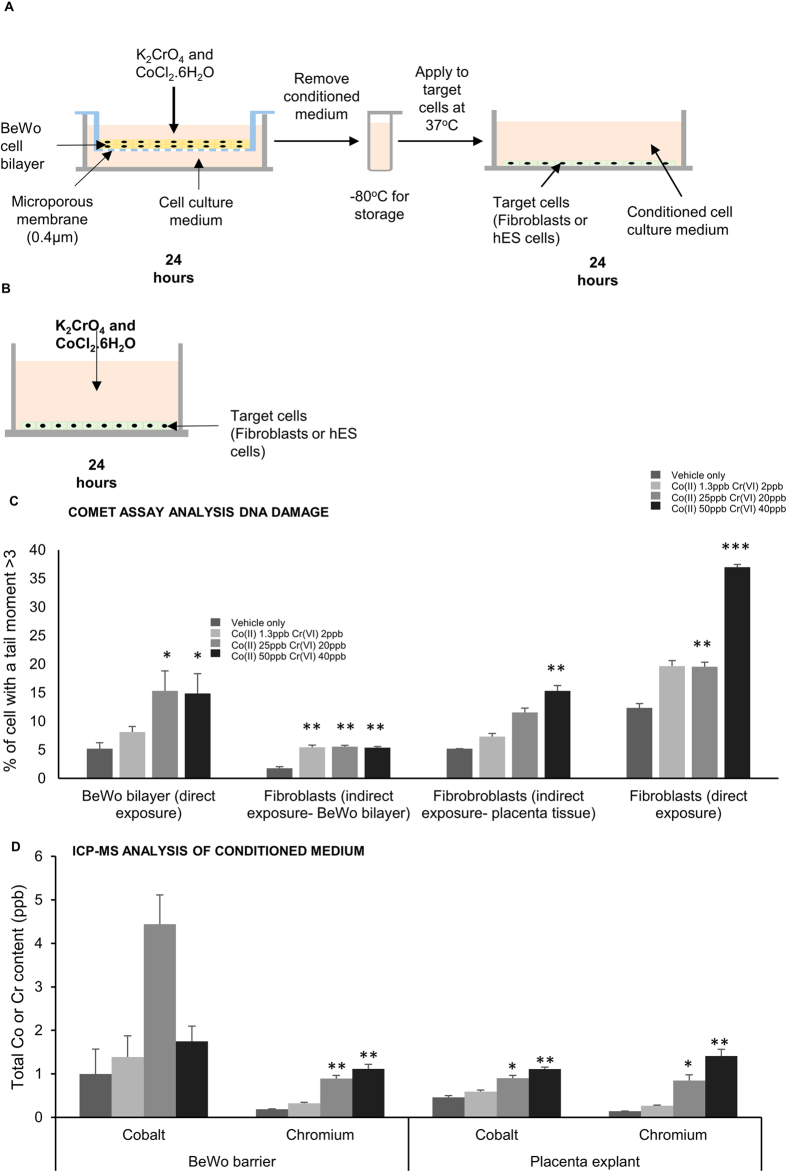
Indirect exposure of fibroblasts to low concentrations of Co and Cr ions induces DNA damage (**a**) Protocol for indirect exposure of target cells (fibroblasts or hES cells) to Co and Cr ions. (**b**) Protocol for direct exposure of target cells (fibroblasts or hES cells) to Co and Cr ions. (**c**) Analysis of DNA damage in the BeWo bi-layer and fibroblast cells using the alkaline comet assay. Three hundred cells (three repetitions of 100) were analysed at random per parameter per experiment. All experiments were repeated three times giving a total of 900 cells scored per parameter. (**d**) Measurement of total Co and Cr concentration in conditioned medium using inductively coupled plasma mass spectrometry (ICP-MS). Conditioned medium was collected from below nine BeWo barriers and from nine placenta explants (taken from three placentae). The data was compared by one-way ANOVA. When a P value of >0.05 was found post hoc Dunnett’s tests were used to compare each treatment group to the negative control. *P > 0.05, **P > 0.01, ***P > 0.001 when compared to the negative control. Centre values represent means. Error bars represent SEM.

It is therefore theoretically possible that a metal-induce bystander effect plays a role in the *in vivo* effects of metal exposure during pregnancy. To investigate this, we prepared a highly simplified laboratory *in vitro* model of the embryo and the developing placenta during the implantation stage of human pregnancy (Fig. 1). Here, human embryonic stem cells (hES cells) would represent a simplified model of the epiblast; a confluent bi-layer of BeWo cells (a placenta trophoblast cell line) grown on a Transwell insert would be a simple model of the trophoblast barrier and the cell culture medium above the trophoblast bi-layer would represent a simple model of the maternal blood. We exposed this trophoblast bi-layer on the apical ‘maternal’ side to low concentrations of Co^2+^ and Cr^6+^ that might be present in the peripheral blood after industrial exposure^16^. We compared the effects of a direct exposure of hES cells to metal, with that of an indirect exposure across the trophoblast bi-layer (Figs 1a,b).

## Results

### Indirect exposure of fibroblasts to low concentrations of Co^2+^ and Cr^6+^ ions induces DNA damage

Previously we have shown that indirect exposure of fibroblast cells to *high* concentrations of Co^2+^ (24 ppb) and/or Cr^6+^ (up to 24 ppb) across a bi-layered trophoblast barrier causes increased DNA damage in the fibroblast cells^13^. In the current experiments it was important to confirm that *lower* concentrations of Co^2+^ (down to 1.3 ppb) and Cr^6+^ (down to 2 ppb) could also induce DNA damage across the trophoblast barrier. This was confirmed (Fig. 1c). In keeping with our previous experiments^13^,^14^, the results suggest that the fibroblast DNA damage is not caused by the metal ions passing through the barrier. Instead the DNA damage may be a response to signalling molecules secreted by the trophoblast cells. This theory is supported by several pieces of evidence. (i) There was significantly increased DNA damage in fibroblasts exposed to the lowest concentration of metal ions indirectly across the barrier but DNA damage did not increase if the fibroblasts were exposed directly to the same concentration (Fig. 1c). (ii) Inductively coupled plasma mass spectrometry (ICP-MS) analysis showed no increase in total Co and Cr in the cell culture medium below barriers exposed to the lowest concentration of metal ions (Fig. 1d) but significantly increased DNA damage was detected in fibroblasts exposed to the lowest concentration of metal ions across the barrier. (iii) Exposure of the barriers to the middle and highest concentrations of metal ions led to a small increase of approximately 1 ppb of Cr in the media below the barrier (Fig. 1d). However there was no increase in DNA damage when fibroblasts were directly exposed either to 1 ppb Cr^6+^ alone (Suppl. Fig. 2) or to 1.3 ppb Co^2+^ and 2 ppb Cr^6+^ (Fig. 1c). The trophoblast cells had significantly increased DNA damage after metal ion exposure (Fig. 1c) however there was no evidence of morphological damage to the barrier or of altered permeability or trans-epithelial resistance (Suppl. Figs 1a–c). The trophoblast barrier of the first trimester placenta is bilayered, and the trophoblast barrier is predominantly monolayered at term.

To investigate whether human placenta tissue would behave in the same way as the bi-layered trophoblast barrier, explants of first trimester placenta were exposed to the same doses of metal, washed three times, and then placed in fresh media to see if they were prompted to release DNA damaging molecules. This media, conditioned by metal exposed placenta, did cause increased DNA damage in fibroblasts when the placenta was exposed to the highest dose of metal (Fig. 1c). Again the metal concentrations in the conditioned media were too low (Co 0.9 ppb and Cr 0.8 ppb for 25:20 ppb Co^2+^:Cr^6+^ and Co 1.1 ppb and Cr 1.4 ppb for 50:40 ppb Co^2+^:Cr^6+^) to cause DNA damage in fibroblasts in a direct exposure (Fig. 1d).

### Indirect exposure of hES cells to Co^2+^ and Cr^6+^ ions induces DNA damage and apoptosis

**Figure 2:**
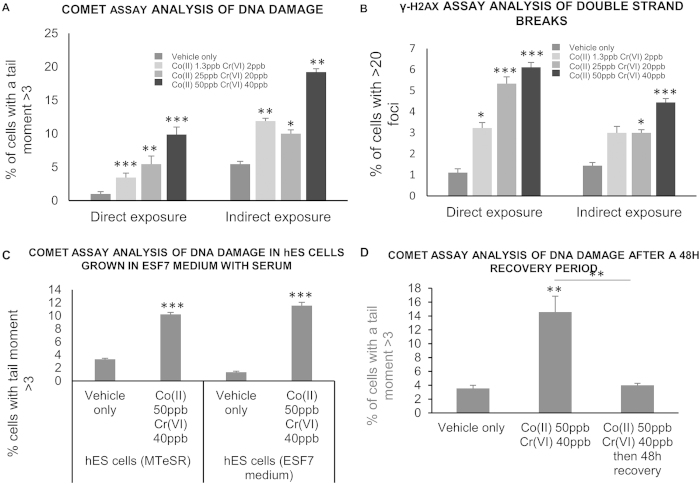
Indirect exposure of hES cells to low concentrations of Co and Cr ions induced DNA damage (**a**) Analysis of DNA damage in directly and indirectly exposed hES cells using the alkaline comet assay. (**b**) Analysis of double strand breaks in directly and indirectly exposed hES cells using the γ-H2AX assay. (**c**) Analysis of DNA damage in indirectly exposed hES cells grown in mTeSR1 (promotes pluripotency) and ESF7 medium + 10% serum (promotes differentiation) using the alkaline comet assay. (**d**) Analysis of DNA damage in indirectly exposed hES cells after a 48 h recovery period using the alkaline comer assay. For all experiments three hundred cells (three repetitions of 100) were analysed at random per parameter per experiment. All experiments were repeated three times giving a total of 900 cells scored per parameter. The data was compared by one-way ANOVA. When a P value of >0.05 was found post hoc Dunnett’s tests were used to compare data from each experimental parameter to the negative control. For (**d**) a Tukeys HSD test was used to compare data from each experimental parameter with that from every other experimental parameter. *P > 0.05, **P > 0.01, ***P > 0.001 when compared to the negative control (unless otherwise indicated). Centre values represent means. Error bars represent SEM.

Indirect exposure of hES cells to Co^2+^ and Cr^6+^ at low doses through a bi-layered trophoblast cell barrier also caused an increase in DNA damage in the hES cells (Figs 2ab). This was seen even at the lowest dose of exposure (1.3 ppb Co^2+^ and 2 ppb Cr^6+^) when Co^2+^ and Cr^6+^ ions do not pass through the barrier (Fig. 1d). The DNA damage included an increase in double strand breaks, as shown by γ-H2AX immunolabelling (Fig. 2b). γ-H2AX foci were counted only in Oct-4 positive cells in order to rule out differentiated cells. A higher proportion of hES cells were damaged if DNA damage was measured by the alkaline comet assay (a measure of single and double strand breaks and alkaline labile sites) compared to the γ-H2AX assay. This suggests that more cells were affected by single strand breaks than by double strand breaks. Preliminary evidence showed an increase incidence of polyploidy in hES cells indirectly exposed to metal through the barrier (Suppl. Fig. 3). Higher levels of DNA damage were detected in indirectly exposed hES cells than in directly exposed cells when measured using the alkaline comet assay (Fig. 2a). This also suggests that DNA damage was not caused by metal ions passing through the barrier. There was no difference in the level of DNA damage as measured with the comet assay in indirectly exposed stem cells which were previously grown in mTeSR1 medium, to encourage pluripotency, and those in ESF7 medium, to encourage differentiation (Fig. 2c). DNA damage reverted to control levels when the conditioned media from Co^2+^ and Cr^6+^ exposed barriers was replaced by normal medium for 48 hours, suggesting that the damage was either repaired or present in cells that had undergone apoptosis (Fig. 2d).

**Figure 3:**
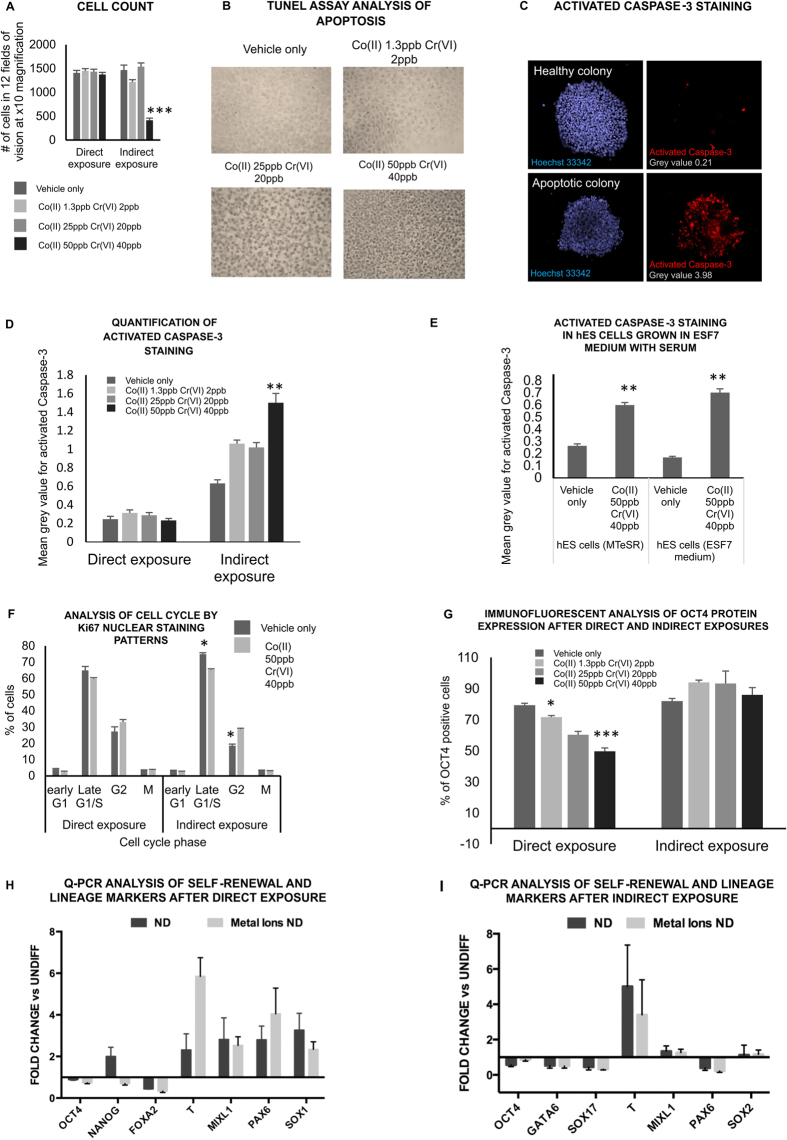
Indirect exposure of hES cells to low concentrations of Co and Cr cause a loss of cells and cell cycle arrest but not differentiation (**a**) Cell count. hES cells were counted *in situ* using an InCell Analyser 1000 high throughput imaging system. All of the cells in 12 fields of vision at x10 magnification in a 96-well tissue culture plate were counted. Twelve wells were prepared per parameter. The experiment was repeated three times. (**b**) TUNEL staining of hES cells after indirect exposure to Co and Cr ions. (**c**) Examples of hES cell colonies stained with activated Caspase-3. ImageJ grey values for activated Caspase-3 staining are shown in grey for each colony. (**d**) Quantification of activated Caspase-3 staining. All of the colonies in one well of a 24-well plate were imaged and the mean grey value calculated using imageJ software. Three wells were prepared for each parameter per experiment. The experiment was repeated in triplicate. (**e**) Analysis of activated Caspase-3 staining in indirectly exposed hES cells grown in mTeSR1 (promotes pluripotency) and ESF7 medium + 10% serum (promotes differentiation). (**f**) Analysis of cell cycle using Ki67 nuclear staining patterns. Three hundred cells (three repetitions of 100) were analysed at random per parameter per experiment. The experiment was repeated three times giving a total of 900 cells scored per parameter. (**g**) Analysis of Oct-4 expression by immunofluorescence. Three hundred cells (three repetitions of 100) were analysed at random per parameter per experiment. The experiment was repeated three times giving a total of 900 cells scored per parameter. The data was compared by one-way ANOVA. When a P value of >0.05 was found post hoc Dunnett’s tests were used to compare each treatment group to the negative control. *P > 0.05, **P > 0.01, ***P > 0.001 when compared to the negative control. Centre values represent means. Error bars represent SEM. (**h**) Q-PCR analysis of self-renewal (OCT4 & NANOG) mesendoderm (T, FOXA2 & MIXL1) and neural (PAX6 & SOX1) markers in stem cells with (black histograms) and without (pale histograms) a direct exposure to metal in E6 medium. These values have been expressed as a ratio or fold change compared to those for cells grown in E8 medium without metal. (**i**) Q-PCR analysis of self-renewal self-renewal (OCT4), endoderm (GATA A6) mesendoderm (T, SOX17 & MIXL1) and neural (PAX6 & SOX2) markers in stem cells with (black histograms) and without (pale histograms) a indirect exposure to metal in E6 medium. These values have been expressed as a ratio or fold change compared to those for cells grown in E8 medium without metal.

In our previous work we saw no significant change in cell number after indirect exposure of fibroblasts or neurones to the signals from metal exposed trophoblast barriers, despite the presence of DNA damage^13^,^14^. However some authors have highlighted a specific vulnerability of hES cells to apoptosis after DNA damage as a result of constitutively active Bax^17^. In the current experiments indirect exposure of the hES cells to the highest concentration of metal, across the trophoblast barrier, caused a significant drop in cell number (Fig. 3a). No corresponding change was seen after a direct exposure to metal (Fig. 3a), again suggesting that the indirect effects of exposure are not due to metal passing through the barrier. The reduction in cell number was accompanied by an increase in positive staining in the TUNEL assay for apoptosis (Fig. 3b). Similarly, there was an increase in caspase-3 activation and nuclear fragmentation, both of which suggest apoptosis (Figs 3c,d). This increase in apoptosis was not noted after direct exposure to metal at these low doses (Fig. 3d). There was also an increase in the proportion of cells in the G2 stage of the cell cycle, as judged by Ki67 nuclear staining patterns, which was statistically significant after indirect but not direct exposure (Fig. 3f). Immunofluorescent staining showed a decrease in the protein expression of the pluripotency marker Oct-4 following direct but not indirect exposure (Fig. 3g). This suggests the possibility of altered cellular differentiation following direct but not indirect exposure. This possibility was give some support by qPCR analysis of mRNA levels (Fig. 3h,i). Here a comparison was made of lineage markers in cells with (black histograms) and without (pale histograms) metal exposure (direct Fig. 3h, indirect Fig. 3i) when the cells were grown in E6 media without TGF β (to encourage gentle differentiation). These data were expressed as a fold change or ratio compared to data for cells without metal exposure when they grown with TGF β (to inhibit cell differentiation). The results showed a similar pattern for cells with and without an indirect exposure to metal (Fig. 3i) but with differences in NANOG, PAX 6 SOX1 and T (brachury) between cells with and without a direct exposure to metal (Fig. 3h).

Overall the results suggest different a biological response of hES cells to direct and indirect exposure to metal across the barrier. After indirect exposure there is evidence for a reduction in cell number which appears to have been due to increased apoptosis and slowing of the cell cycle resulting from the DNA damage. However, no differentiation was observed. After direct exposure there was evidence for similar DNA damage with altered differentiation and no loss of cells or cell cycle arrest.

### Barrier cells transmit indirect response of hES cells to metal toxins via TNF-α relaese

**Figure 4:**
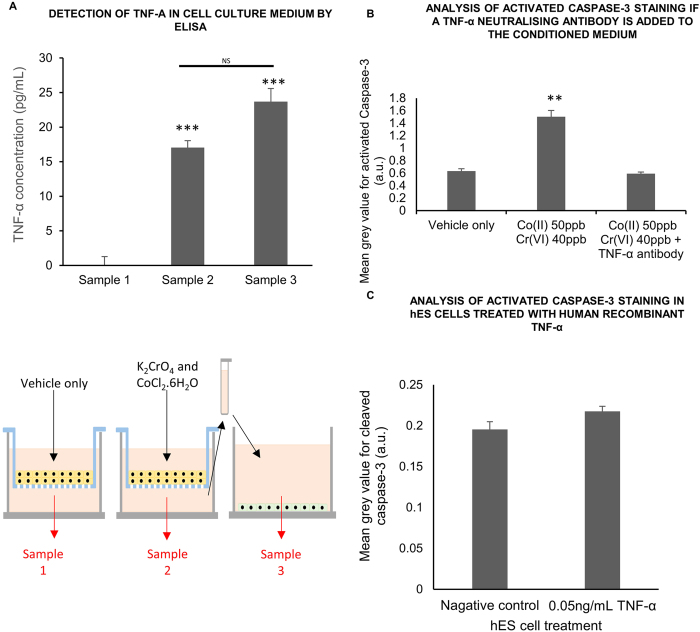
Cytokine secretion from BeWo bilayers after Co and Cr ion exposure (**a**) Measurement of TNF-α concentration in cell culture medium below unexposed BeWo bilayers (sample 1), below BeWo bilayers exposed to Co(II) 50 ppb Cr(VI) 40 ppb (sample 2) and bathing hES cells after indirect exposure (sample 3) by ELISA. The experiment was repeated on nine separate occasions and one aliquot of cell culture medium was tested for each sample (1, 2 and 3) per experiment. (**b**) Analysis of activated Caspase-3 immunofluorescent staining in hES cell colonies indirectly exposed to Co and Cr ions in the presence of a TNF-α neutralizing antibody. (**c**) Analysis of activated Caspase-3 immunofluorescent staining in hES cell colonies indirectly exposed to 50 ng/mL human recombinant TNF-α. For (**b**) and (**c**) ninety hES cell colonies (thirty hES cell colonies on each of three glass coverslips) were analysed at random per experimental parameter per experiment. The experiment was repeated on three separate occasions giving a total of 270 hES cell colonies scored per parameter. In (**a**) and (**b**) the data was compared by one-way ANOVA. When a P value of >0.05 was found post hoc Tukey’s HSD tests were used to compare data from each experimental parameter with that from every other experimental parameter. For (**c**) an unpaired students-*t* test was used. NS not significant *P > 0.05, **P > 0.01, ***P > 0.001 when compared to the negative control (unless otherwise indicated). Centre values represent means. Error bars represent SEM.

The difference between the survival and differentiation of indirectly and directly exposed cells, both of which were DNA damaged, suggested the possibility that a second factor that was released from the barrier in addition to one that caused DNA damage. In order to explore this, a semi-quantitative screen of 6 cytokines was performed, choosing cytokines that were known to be released from metal toxin exposed barriers (corneal) and including TGF-β and TNF-α, which had been identified as influencing the bystander effect^14^,^15^,^17^. There was a significant increase of TNF α but no change in TGF-β, MCP1, IL-I beta, IL-2 IL-8 or IL-6 in the conditioned media below metal exposed barriers. The increase in TNF-α was confirmed quantitatively (Fig. 4a). Measurements were made of the media around hES cells, which had been exposed to the conditioned media below barriers, to test whether the hES cells themselves may have secreted TNF-α, as well as the trophoblast barrier. Although the levels were higher, this was not significant (Fig. 4a). Applying a neutralising antibody to TNF-α reduced activated caspase-3 staining to control levels (Fig. 4b). However exposing hES cells to human recombinant TNF-α did not increase activated caspase-3 staining (Fig. 4c). These results suggest that most of the TNF-α is secreted from the trophoblast barrier rather than the hES cells themselves and that TNF-α is required but not sufficient to cause apoptosis in the hES cells after this indirect exposure to metal across the trophoblast barrier.

### DNA damaging signals are transduced via connexin 43 gap junctions

**Figure 5:**
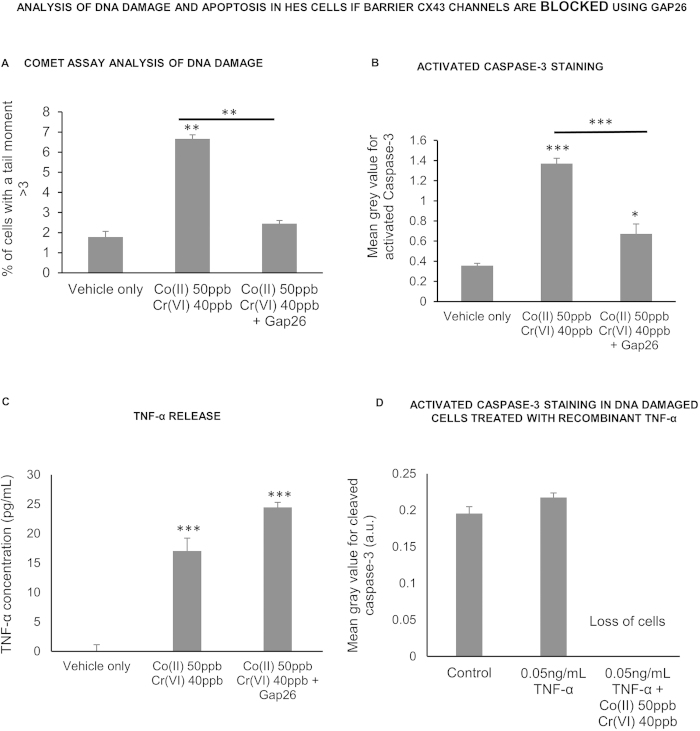
Influence of connexin 43 channels within the BeWo bilayer on bystander-like signalling (**a**) Analysis of DNA damage in hES cells indirectly exposed to Co and Cr ions across a Gap26 treated BeWo bilayer using the alkaline comet assay. Three hundred cells (three repetitions of 100) were analysed at random per parameter per experiment. All experiments were repeated three times giving a total of 900 cells scored per parameter. (**b**) Analysis of activated Caspase-3 immunofluorescent staining in hES cell colonies indirectly exposed to Co and Cr ions across a Gap26 treated BeWo bilayer. (**c**) Analysis of TNF-α concentration in cell culture medium below BeWo bilayers exposed to vehicle only or Co(II) 50 ppb Cr(VI) 40 ppb or Co(II) 50 ppb Cr(VI) 40 ppb plus Gap26. Conditioned medium from below nine BeWo bilayers was tested per experimental parameter. (**d**) Analysis of activated Caspase-3 immunofluorescent staining in healthy or DNA damaged hES cell colonies treated with human recombinant TNF-α. For (**b**) and (**c**) ninety hES cell colonies (thirty hES cell colonies on each of three glass coverslips) were analysed at random per experimental parameter per experiment. The experiment was repeated on three separate occasions giving a total of 270 hES cell colonies scored per parameter. The data was compared by one-way ANOVA. When a P value of >0.05 was found post hoc Tukey’s HSD tests were used to compare data from each experimental parameter with that from every other experimental parameter. *P > 0.05, **P > 0.01, ***P > 0.001 when compared to the negative control (unless otherwise indicated). Centre values represent means. Error bars represent SEM.

In our previous work we showed that connexin 43 gap junction and hemichannels within the trophoblast barrier were key elements in the signalling from barrier to fibroblast^13^,^14^. In the current work the DNA damaging signalling to hES cells was prevented by applying an antagonist of connexin 43, the connexin mimetic peptide Gap 26, to metal exposed barriers (Fig. 5a). This also prevented the increase in activated caspase-3 staining in the hES cells (Fig. 5b). However it did not alter the secretion of TNF-α from the barrier (Fig. 5c), which also suggests that TNF-α exposure of hES cells alone was not sufficient to induce apoptosis and that a DNA damaging signal might also be required. To test this, the hES cells were directly exposed to both human recombinant TNF-α and Co^2+^ and Cr^6+^ ions, neither of which induced apoptosis staining on their own (Figs 3b and 4c) although direct exposure to metal ions did cause DNA damage in hES cells (Figs 2a,b). The combination of TNF-α and metal resulted in a dramatic increase in cell death (Fig. 4d), in keeping with the idea that apoptosis was caused by a combination of TNF-α and DNA damage.

**Figure 6:**
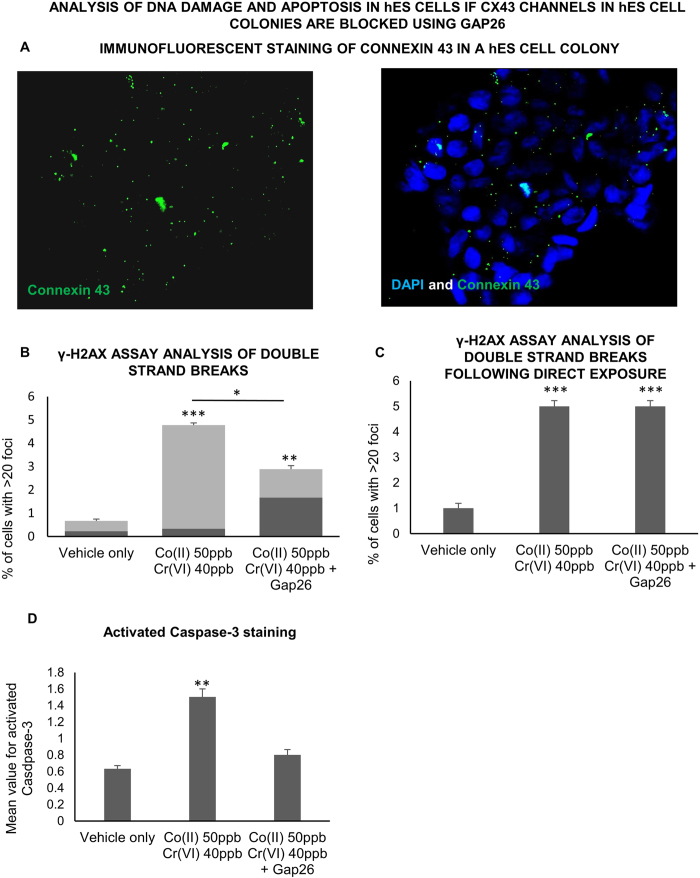
Influence of connexin 43 channels within the hES cell colonies on bystander-like signalling (**a**) Immunofluorescent staining of connexin 43 in a hES cell colony. (**b**) Analysis of double strand breaks in indirectly exposed hES cell colonies with and without treatment of the colony with Gap26. Light grey: % of cells that have > 20 gamma H2AX foci and who’s three nearest neighbors also have > 20 foci. Dark grey: % of cells that have > 20 gamma H2AX foci and who’s three nearest neighbors do not have > 20 foci. (**c**) Analysis of double strand breaks in directly exposed hES cell colonies with and without treatment of the colony with Gap26. For (**b**) and (**c**) Three hundred cells (three repetitions of 100) were analysed at random per parameter per experiment. All experiments were repeated three times giving a total of 900 cells scored per parameter. (**d**) Analysis of activated Caspase-3 immunofluorescent staining in hES cell colonies indirectly exposed to Co and Cr ions with and without treatment of the colony with Gap26. Ninety hES cell colonies (thirty hES cell colonies on each of three glass coverslips) were analysed at random per experimental parameter per experiment. The experiment was repeated on three separate occasions giving a total of 270 hES cell colonies scored per parameter. The data was compared by one-way ANOVA. When a P value of >0.05 was found post hoc Tukey’s HSD tests were used to compare data from each experimental parameter with that from every other experimental parameter. *P > 0.05, **P > 0.01, ***P > 0.001 when compared to the negative control (unless otherwise indicated). Centre values represent means. Error bars represent SEM.

It was noted that hES cells with very high levels of double strand breaks were present in clusters of adjacent cells rather than being distributed evenly throughout cell colonies (Suppl. Fig. 4). This suggested that the hES cells might have sent DNA damaging signals to each other via gap junctions. Immunolabelling of connexin 43 in hES cell colonies confirmed the presence of plaque like gap junctions between cells (Fig. 6a). Applying Gap 26 to the indirectly exposed hES cells reduced the overall level of double strand breaks, as well as the percentage of cells with double strand breaks whose nearest neighbours also had double strand breaks (Fig. 6b). However it actually increased the percentage of isolated cells with high levels of double strand breaks, whose nearest neighbouring cells were without double strand breaks (Fig. 6b). This is in keeping with the possibility that the cells with high levels of double strand breaks could have sent DNA damaging signals to neighbouring cells through gap junctions/hemichannels. Treatment of directly exposed hES cell colonies with Gap 26 did not reduce the level of double strand breaks, again highlighting important difference between indirect and direct metal exposure (Fig. 6c). Gap 26 also reduced the percentage of apoptotic colonies after indirect exposure (Fig. 6d).

## Discussion

We have developed a novel albeit simplified *in vitro* model for early trans-placental fetal signalling that combines BeWo placenta barrier culture with human embryonic stem cell culture. Using this model we have identified a fundamental difference between the responses of hES cells to physiologically relevant^6^,^8^ levels of toxic metals (Co^2+^ and Cr^6+^) according to whether the cells are exposed directly or exposed indirectly through a bi-layer of trophoblast cells (as is seen during the first trimester of pregnancy). The direct exposure of hES cells to Co^2+^ and Cr^6+^ led to DNA damage, mostly as single strand breaks and alkaline labile sites but including some double strand breaks, but without cell death or an altered cell cycle. In contrast the indirect exposure across the trophoblast cell bi-layer led to DNA damage, which recovered and which was associated with increased cell death and a stall of the cell cycle at G2.

Our simplified model of the interrelationship between trophoblast and embryonic stem cell, if relevant, would apply to the early stages of human pregnancy when the implanting blastocyst consists of a trophoblast shell surrounding an inner cell mass. It should be noted that although hES cells are derived from the inner cell mass of the blastocyst they express cell and molecular properties of primitive ectoderm and epiblast. The potential for gap junction intercellular communication (GJIC) between the different cell types within the *in vitro* model is similar to the implantation stage of pregnancy *in vivo*. Connexin 43 gap junctions exist between BeWo cell to BeWo cells and between hES cell to hES cell but the BeWo cells are not coupled to hES cells via gap junctions. Similarly, gap junctions interconnect the trophoblast cells in the developing placenta and also the cells of the epiblast^18^,^19^.

We show that the mechanism that accounts for the difference in hES cell response to an indirect versus a direct exposure of metal ions is due to bystander-like signalling not just within the trophoblast bi-layer but also within the hES cell colonies. The trophoblast bi-layer releases at least two signals, a DNA damage signal and TNF-α. Both of these signals are required for cell death and neither is sufficient alone.

TNF-α is a cytokine with pleiotropic effects, with critical regulatory roles in cell proliferation, differentiation and programmed cell death. TNF-α and its receptor are expressed in the placenta, uterus and the embryos of both humans and experimental animals^20^,^21^,^22^. High levels of TNF-α are associated with miscarriage^23^,^24^,^25^. Increased TNF-α has been detected in the placenta in cases of recurrent miscarriage in humans^26^. Similarly, diabetic mice have a decreased pregnancy rate associated with increased TNF-α protein and mRNA in the uterus^27^. TNF-α has also been implicated as a mediator of detrimental stimuli. Lipopolysaccharide induced foetal resorption is TNF-α mediated^28^. Furthermore, elevated TNF-α mRNA and protein, as well as TNFR1 mRNA levels have been detected in the uterine epithelium and stroma and in the giant and spongiotrophoblasts of the placenta of mice with miscarriage induced by DNA damaging cyclophosphamide^29^. The current work has shown that exposure of a trophoblast bi-layer to metals stimulates TNF-α secretion. This could provide a possible mechanism for the increased risk of foetal resorption and miscarriage in mothers exposed to some toxins during pregnancy.

The mechanism for indirect metal exposure also depends on gap junctions in the trophoblast bi-layer and in the hES cell colonies. Blockade of gap junctions at either site reduced DNA damage and cell death. There is a wealth of evidence to suggest that gap junctions are important conduits for cell signalling molecules promoting either cell death or survival, which has been reviewed elsewhere^30^. The decision between the two opposite biological outcomes depends on the status of the cell that receives the signal and its environmental context. As a further emphasis on the difference between direct and indirect metal exposure, blockage of gap junctions within the hES cell colonies does not reduce DNA damage in directly exposed cells.

The metal ion-induced signalling between the trophoblast bi-layer and hES cells is similar to the bystander phenomenon described in radiation biology. The term radiation-induced bystander effect is used to describe the radiation-induced biological changes that occur in non-irradiated cells, including DNA damage and cell death^31^. The bystander signal(s) that passes from one cell to another is unknown despite years of investigation^32^. However, there are several indicators that signalling at a distance can occur *in vivo* and therefore potentially from a trophoblast layer to a developing foetus. Currently there is a consensus that soluble clastogenic factors, including extracellular free radicals, which cause DNA damage, can reach different tissues within an organism from the circulation of blood cells that are exposed to radiation^33^. Like the effects in our experiment, the long range damage *in vivo* can be reduced when expression of connexin 43 is inhibited^34^. There are also studies which suggest that there are secreted molecules that are induced by mitochondrial stress and signal to distal tissues called mitokines^35^.

In general there is little information on the induction of a bystander signal in embryos or foetuses *in vivo* which probably reflects the technical difficulty in the design and execution of such an experiment. Bystander effects are considered likely to occur post-implantation^36^. Wang *et al.*^37^ for example demonstrated a radiation induced bystander effect for cultured limb bud cells which altered cell proliferation and chondrogeneisis which was inhibited again by blocking gap junction communication. These authors point to the potential relevance of this to development as gap junction communication and intercellular communication are both involved in the development of the fetus.

The significance of the increased apoptosis and DNA damage in the stem cells in our model system after indirect exposure to metals is not known. Our model is limited and is a highly simplified representation of an *in vivo* exposure. Animal experiments have demonstrated a decrease in implantation sites and number of foetuses, and an increase in foetal malformations, if the mother is exposed to metal during pregnancy^7^,^38^,^39^. Epidemiological studies have shown an association between some maternal metal exposures and lymphocyte DNA damage and retinoblastoma in offspring^5^,^6^. A recent meta-analysis has shown an association between occupational Cr poisoning and risk of spontaneous abortion in China^1^. The current view is that fetal damage is caused by the metals passing through the placental barrier and reaching the fetus. However, in our model system, it is the indirect effects of exposure across the placental trophoblast barrier, rather that the direct effects of exposure, that are more in keeping with the effects noted *in vivo*. Further experiments to investigate the possibility of signalling between trophoblast and embryonic cells are warranted.

## Materials and Methods

### Cell culture

Primary human BJ skin fibroblasts (LGC Promochem UK) were maintained in T-75 flasks (Corning) in Minimal Essential Medium (Sigma-Aldrich) supplemented with 10% (v/v) foetal bovine serum (Gibco), 1% (v/v) antibiotic antimitotic solution (containing 1000 units penicillin, 10 mg streptomycin and 25 μg amphotericin B per mL, Sigma Aldrich), 0.1 mg/mL sodium pyruvate, 0.02M HEPES buffer and 2 mM L-glutamine (all from Sigma-Aldrich) at 5% CO_2_ and 37 °C. The medium was changed every 2–3 days. The cells were passaged at a ratio of 1:2 upon reaching 80% confluence using 0.25% Trypsin-EDTA solution (Sigma Aldrich) for 2 minutes at 37 °C. For experiments the fibroblast cells were plated into 12-well plates at a density of 50,000 cells per well.

Colonies of H9 human embryonic stem cells^40^ (WiCell (Madison, WI)) were grown on irradiated mouse embryonic fibroblast feeder layers. The colonies were maintained in Knock Out Dulbecco’s Modified Eagles Medium (KO-DMEM), supplemented with 10% (v/v) knock out serum replacement, 1% (v/v) non- essential amino acids, 1% (v/v) 200 mM L- glutamine, 1% (v/v) penicillin-streptomycin (10,000 units penicillin and 10,000 μg streptomycin per mL) (Gibco), 0.1 mM β- Mercaptoethanol (Sigma Aldrich) and fibroblast growth factor 2 (FGF2, 20 ng per mL). The cells were passaged at a ratio of 1:4 upon reaching 80% confluence (approximately every 3 days) using 1 mg/mL collaganase IV (Gibco) plus 10 μM ROCK inhibitor Y27632 (Millipore) in KO-DMEM for 45 minutes at 37 °C and 5% CO_2._ For experiments, H9 cells were plated into multi-well plates in a feeder cell free system. The surfaces were coated with Matrigel^TM^ (hESC qualified matrix, BD Biosciences) and the H9 cells cultured in mTeSR1^TM^ defined maintenance medium for hESCs (Stem Cell Technologies), supplemented with 1% (v/v) penicillin-streptomycin (10,000 units penicillin and 10,000 μg streptomycin per mL) (Gibco). Cell colonies were grown for up to six days before exposure to Co^2+^ Cr^6+^. In some cases the H9 cells were allowed to differentiate in multi-well tissue culture plates prior to or following Co^2+^ Cr^6+^ exposure. Here, the cell culture surfaces were coated with 0.1% gelatine solution (EmbryoMax) and the cells maintained in ESF7 cell culture medium (Cell Science and Technology Institute, Sendai, Japan) supplemented with 10% foetal bovine serum. The morphology of the H9 colonies was examined using an inverted phase contrast microscope prior to Co^2+^ Cr^6+^ exposure to ensure that they were beginning to differentiate.

BeWo cells^41^ (gift from Dr. A. Schwartz of Washington University, St Louis, MO) were cultured in T-75 flasks (Corning) in Dulbecco’s Modified Eagle Medium- F12-HAM (Sigma) enriched with 10% (vlv) foetal bovine serum (Gibco), 1% L-glutamine (200 mM solution) and 1% (v/v) antibiotic antimitotic solution (containing 1000 units penicillin, 10 mg streptomycin and 25 μg amphotericin B per mL) (Sigma Aldrich) at 37 °C and 5% CO_2_. The cells were passaged at a ratio of 1:10 upon reaching 80% confluence (approximately every seven days) using 0.25% Trypsin-EDTA solution (Sigma Aldrich) for 5 minutes at 37 °C. BeWo bi-layer barriers were prepared as reported previously^13^,^14^. Here BeWo cells were seeded onto porous Transwell permeable supports (Corning (0.4 μm) at a density of 112,000 cells in 0.5 mL of medium and grown for seven days with media changes on days 2, 5 and 6.

### Placental tissue

First trimester human placenta (8–9 weeks gestation) was obtained with written informed consent following elective surgical termination of pregnancy with local research ethics committee approval. The clinical sample collection was carried out in accordance with the guidelines approved by the Ethics Committee of Southwest-Bristol. 0.5 cm^3^ explants of the villous tissue were dissected and maintained in 12-well plates for a maximum of 48 h. Each explant was bathed in 1.5 mL of trophoblast cell culture medium containing a 1:1 ratio of Minimal Essential Medium and F12 Nutrient Mixture (Ham) (Gibco), supplemented with 10% (v/v) foetal bovine serum (Gibco), 1% (v/v) L-glutamine (200 mM solution, Sigma-Aldrich), 1% (v/v) antibiotic antimitotic solution (containing 1000 units penicillin, 10 mg streptomycin and 25 μg amphotericin B per mL, Sigma Aldrich) and 0.5% (v/v) gentamicin (10 mg/mL solution, Gibco).

### Direct and indirect exposures to Co^2+^ Cr^6+^

Cells were directly or indirectly exposed to 1.3 ppb, 25 ppb and 50 ppb Co^2+^ (cobalt chloride (CoCl_2_.6H_2_O)) combined with 2 ppb, 20 ppb and 40 ppb Cr^6+^ (potassium dichromate (K_2_CrO_4_) respectively. For vehicle only controls distilled water (with no metal ions) was added to the cell culture medium. Fibroblast cells or hES cells were directly exposed by adding dissolved metal salts into the cell culture medium bathing either. They were harvested and analysed after 24 hours of direct exposure. Indirect exposures were made adding dissolved metal salts into the upper chamber of the Transwell inserts above the BeWo barriers. After 24 hours the cell culture medium from the lower chamber was collected and stored at −80 °C before being warmed and applied to fibroblasts or hES cells. The cells were harvested and analysed after 24 hours of indirect exposure (Suppl. Fig. 6). For placental explant exposures the metal salts were dissolved into the culture medium bathing the placental tissue. After 24 hours the medium was removed and the cultures washed five times in PBS. The explants were then maintained in fresh culture medium for 24 hours. At this point the culture medium was collected and stored at −80 °C before being warmed and applied to fibroblasts. All cells were maintained at 37 °C and 5% CO_2_ throughout the direct and indirect exposure protocols.

### Reagents used to investigate signaling

Gap 26 connexin mimetic peptide (150 μM) (VCTDKSFISHUR) (Tocris Biosciences #1950) was used to specifically inhibit connexin 43 gap junctions in the BeWo barrier and H9 cell colonies. Anti-TNF-α antibody (0.1 μg/mL) (Abcam #ab9635) was used to neutralise TNF- α in conditioned cell culture medium. Recombinant human TNF-α (50 ng/mL) (Gibco #PHC3015) was added directly to hES cell cultures.

### Analysis of cells

In all cases cells were analysed by a blinded researcher who did not know the treatment parameter to which each sample belonged.

The alkaline comet assays were performed as previously described^41^. Cells were visualised under the   40 objective using a fluorescence microscope (Olympus BX-50) with an excitation filter of 515–560 nm and barrier filter of 590 nm. Image analysis software (COMET III, Perceptive Instrument) was used to quantify the tail moment (a product of comet length and tail intensity) of individual cells. Three hundred cells (three repetitions of 100) were analysed at random per experiment and experiments were repeated on three separate occasions, giving a total of 900 cells analysed per parameter. Cells with a tail moment greater >3 were considered to have a high level of DNA damage (Suppl. Fig. 5).

For immunofluorescence, cells were fixed with 4% paraformaldehyde (Sigma Aldrich) in PBS for 15 minutes at 4 °C. Cells were permeabilised by washing three times in PBST; PBS supplemented with 0.1% (v/v) Triton x-100 (Sigma-Aldrich), for 5 minutes. To block non-specific antibody binding, 1% bovine serum albumin (Sigma- Aldrich) (w/v) in PBS was applied for one hour at room temperature. The primary antibodies were applied overnight at 4 °C. The secondary antibodies were applied for one hour at room temperature (See Suppl. Table 1). Cell nuclei were counterstained using 1 μg/mL Hoechst-33342 for 5 minutes. Stained cells were viewed using an Olympus BX14 microscope equipped with single band pass filters and an AX10 Cam MRm camera (Zeiss). ISIS imaging software (Metasystems) was used to capture and store images. For γ-H2AX, Oct-4 and Ki67 staining three hundred cells (three repetitions of 100) were analysed at random per experiment and experiments were repeated on three separate occasions, giving a total of 900 cells analysed per parameter. For the γ-H2AX assay the number of foci per cell was counted in Oct-4 positive cells only (Suppl. Fig. 6) under the x100 objective. Cells with >20 foci were considered to have a high level of DNA damage (Suppl. Figure 7). The graphs show the mean percentage of cells with >20 foci for each experimental parameter. For Ki67 the nuclear expression pattern of Ki67 protein was analysed at x40 magnification and was used to classify the cell cycle stage for each cell (Suppl. Figure 8). For activated caspase-3 staining an average of 90 colonies (three repetitions of approximately 30 colonies per cover slip) were analysed per experiment parameter. The experiments were repeated on three separate occasions. Images were captured using the   10 objective. Colonies were classified as normal or apoptotic according to the extent of nuclear fragmentation and activated caspase-3 staining (Fig. 4d). Individual cells were not scored because hES cell colonies are densely populated and caspase-3 is expressed in the cytoplasm.

Metaphase spreads were prepared with cells *in situ* on glass cover slips. Cells were treated with Karyomax Colcemid^TM^ (Life Technologies) for 6 hours followed by 0.075M KCl (Sigma Aldrich) 30 minutes at room temperature. The cells were fixed by drop-wise addition of 6:1 methanol:acetic acid. The KCL and fixative were removed and the cells incubated in 3:1 and then 6:1 methanol:acetic acid for 2 minutes each. Cover slips were air dried and allowed to age overnight at room temperature. Chromosomes were stained with 1 μg/mL Hoechst-33342 for 5 minutes and viewed with a Olympus BX14 microscope ( 100 objective). All metaphases were scored on three cover slips for each experimental parameter(Suppl. Fig. 3).

Cell counts were performed *in situ* in 96-well plates. Cells were fixed in 4% paraformaldehyde and stained with 1 μg/mL Hoechst-33342 for 5 minutes. All cells in 12 fields of view ( 20) in each well were counted using an InCell Analyser 1000 high throughput cellular imaging and analysis system (Amersham Biosciences). 12 wells were examined per treatment and the experiment was repeated three times.

The TUNEL assay was carried out using a TACS TdT-DAB *in situ* apoptosis detection kit (Trevigen #4810-30-k) in accordance with the manufacturer’s instructions. Images of hES cell colonies were captured and were assessed visually for TUNEL positive staining.

### Analysis of the barrier

BeWo barriers were fixed in formal saline for 20 minutes at 4 °C and processed for paraffin wax histology. The barriers were cut from the Transwell inserts using a scalpel blade and sandwiched between two layers of 1% agarose gel. The agarose and barrier were then dehydrated in graded ethanol (Sigma Aldrich) embedded in paraffin wax and sectioned at 5 μM. Sections were stained with haematoxylin and eosin (Sigma Aldrich) and visualised using a bright field microscope (Zeiss Axio Imager) connected to a camera (QImaging MicroPublisher 3.3 RTV). Each barrier was examined along its entire length and cells were classified as being in layer 1 (in contact with the polyester support), layer 2 or layer 3.

Transepithelial electrical resistance measurements was measured with an EVOM volt-ohmeter connected to a 12 mm Endohm unit (World Precision Instruments, Sarasota FL).

Barrier integrity was assessed by measuring the passage of Fluorescein isothiocyanate conjugate bovine serum albumin (FICT-BSA, Sigma Aldrich) from the upper chamber to the lower chamber (across the barrier) at 37 °C and 5% CO_2_. 1.5 mL of phenol red free medium containing BSA (1 mg per mL) was added to the bottom chamber. After 1 hour, 300 μL sample of medium was taken from the bottom chamber and placed into the wells of a 96-well plate. The 300 μL sample was replaced with 300 μL of DMEM plus BSA (0.1 mg per mL). This process was repeated at 2 hours and 3 hours. The level of FITC-BSA was detected with a fluorescent plate reader (488 nm).

Three BeWo barriers were analysed for each experimental parameter (e.g. control, low, medium and high dose of metal) per experiment. Each experiment was repeated on three separate occasions.

### Analysis of the cell culture media

The concentration of Co and Cr in the culture medium was measured with Inductively coupled plasma mass spectrometry (ICP-MS).

A Multi-Analyte ELISArray Kit (Qiagen) was used to detect TNF-α, TGF-β, IL-6, IL-8, IL-2, MCP1 and IL1-β in cell culture medium in accordance with the manufacturer’s instructions.

A Single-Analyte ELISArray kit (Qiagen) was used to measure TNF-α concentration in cell culture medium against an antigen standard curve in accordance with the manufacturer’s instructions.

In all cases the cell culture medium from below three BeWo barriers was analysed per experimental parameter (e.g. control, low, medium and high dose of metal) per experiment. Each experiment was repeated on three separate occasions.

### Statistical analysis

Statistical analysis was performed with IBM SPSS statistics 21 software. The Shapiro-Wilk test was used to assess if data was normally distributed. Student’s *t* tests were used for normally distributed data where only two experimental groups were compared. One-way analysis of variance (ANOVA) was used for normally distributed data where three or more experimental groups were compared. When a P value of >0.05 was found post hoc tests were performed. Dunnett’s test was used for data where several treatment groups were compared to the negative control. Tukey’s HSD test was used for data where experimental groups were compared to each other in all possible combinations. In some cases, where the data was not normally distributed, transformations were applied to the data to ensure that variance between groups showed less than a 5-fold difference. A natural log transformation was applied to cell count data. An arcsine square root transformation was applied to data which represented a proportion of cells to help fulfil the ANOVA assumption that the dependent variable is continuous. If the data failed to meet the assumption of homogeneity of variance, Student’s *t* tests were performed with Welch correction to compare two experimental groups. Similarly, Welch ANOVA was used for multiple comparisons. When a P value of >0.05 was found post hoc Games-Howell tests were performed^42^,^43^.

## Additional Information

**How to cite this article**: Jones, A. J. *et al.* Evidence for bystander signalling between human trophoblast cells and human embryonic stem cells. *Sci. Rep.* **5**, 11694; doi: 10.1038/srep11694 (2015).

## Supplementary Material

Supplementary Information
