# Supplementary material for: Evidence for bystander signalling between human trophoblast cells and human embryonic stem cells
Source: Sci Rep. 2015 Jul 14;5:11694. doi: 10.1038/srep11694 (PMC4501009; doi:10.1038/srep11694)
Supplement: Supplementary Information [file srep11694-s1.pdf]

Evidence for bystander signalling between human trophoblast cells and human embryonic stem cells

Anna J Jones[1] Paul J Gokhale[2] Tom Allison [2] Barry Sampson[3] Sharan Athwal [4] Simon Grant [4] Nicholas DK Allen[5] Peter W Andrews [2] C Patrick Case [1*]

1. Musculoskeletal Research Unit, School of Clinical Sciences (North Bristol), University of Bristol, Bristol BS10 5NB
2. Centre for Stem Cell Biology, Department of Biomedical Science, The University of Sheffield, Sheffield S10 2TN
3. GMO 02 Medical Oncology Block, Medical Oncology Charing Cross Campus London SW7 2AZ,
4. Department of Obstetrics, Southmead Hospital, Bristol BS10 5NB
5. Cardiff School of Biosciences, The Sir Martin Evans Building, Museum Avenue, Cardiff, CF10 3AX

*0117 4147927 **c.p.case@bristol.ac.uk**

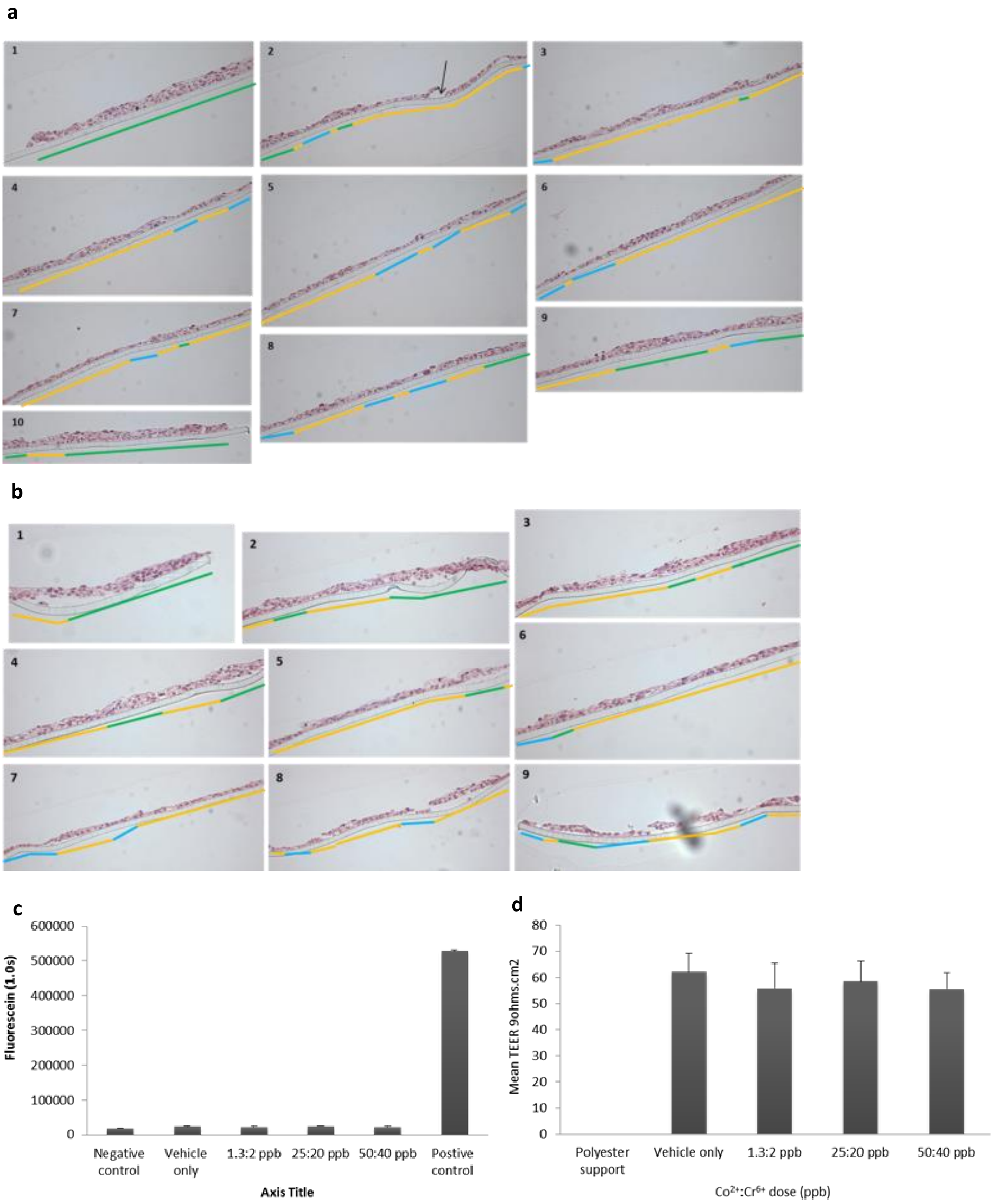

Suppl. Figure 1 The BeWo barrier is not damaged by metal exposure a) H&E stained cross section through the entire length of an unexposed control BeWo barrier. Bi-layered areas underlined in orange, tri-layered areas underlined in green and monolayered areas underlined in blue. The barrier is predominantly (approximately 70%) bi-layered. Tears in the barrier are due to processing steps (including cutting the membrane from the Transwell insert with a scalpel blade) and appear in unexposed barriers as well as barriers exposed to metal. b) H&E stained cross section through the entire length of BeWo barrier exposed to the highest concentration of metal. c) Passage of FIT-C labelled bovine serum albumin through unexposed control BeWo barriers and metal exposed barriers. d) Trans epithelial electrical resistance measurements from unexposed control BeWo barriers and metal exposed barriers. Centre values represent means. Error bars represent SEM.

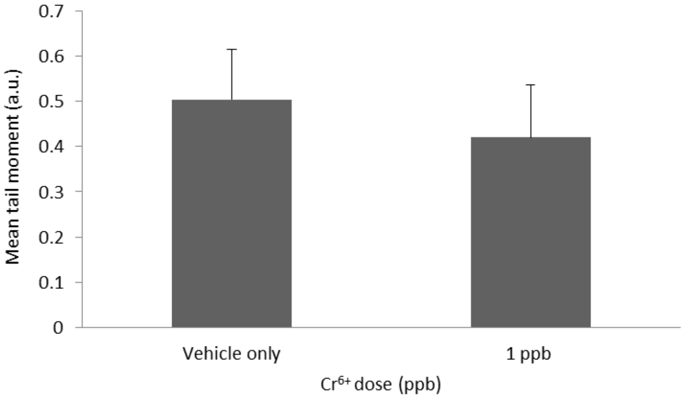

Suppl. Figure 2 Direct exposure of fibroblasts to 1ppb Cr^{6+} does not cause an increase in comet assay mean tail moment. Centre values represent means. Error bars represent SEM.

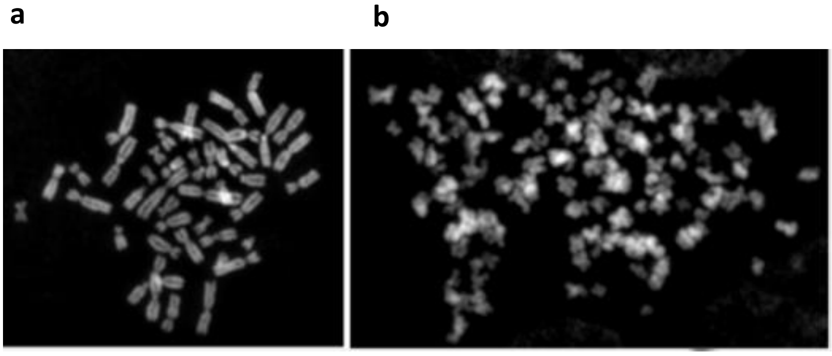

Suppl. Figure 3 Chromosome counts in hES cells following indirect metal exposure across a BeWo barrier. a) hES cell metaphase spread with normal chromosome number (46) b) hES cell metaphase spread with obvious increase in chromosome number following indirect exposure to 50:40ppb Co^{2+}:Cr^{6+}. 3.4% (7 out of 207) of indirectly exposed hES cells showed evidence of polyploidy compared to 0% of control cells.

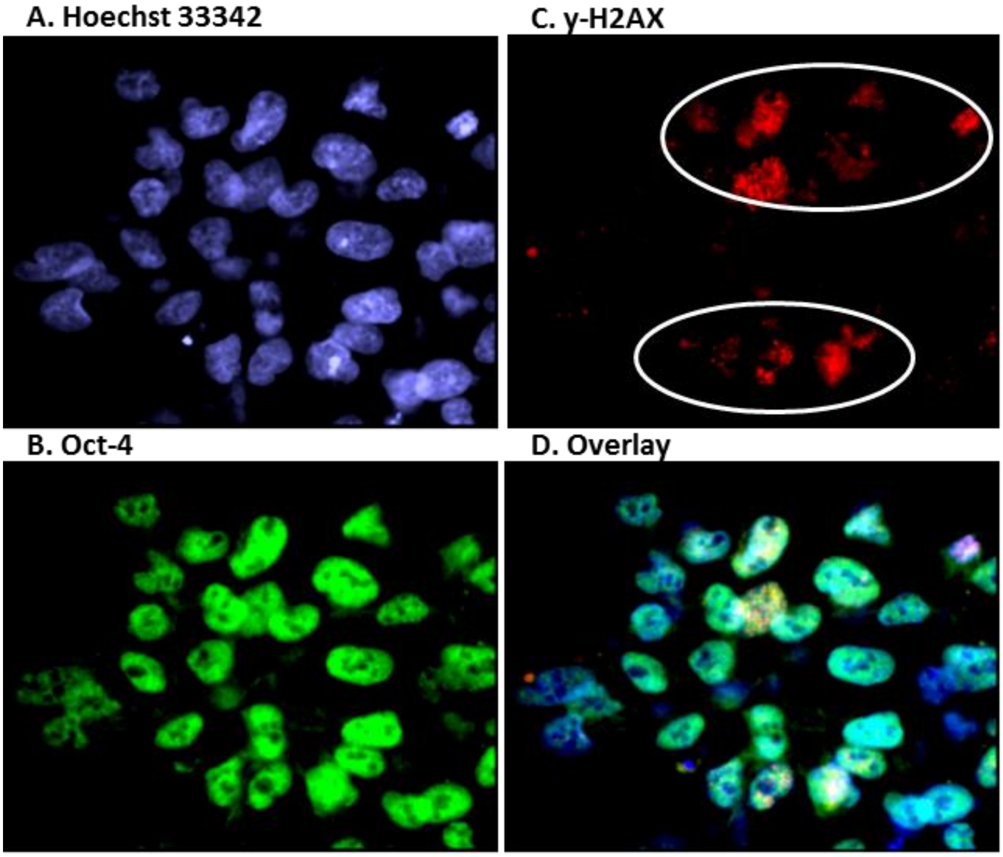

Suppl. Figure 4: Clustering of γ-H2AX positive cells

The image shows H9 cells following indirect exposure to vehicle only or 50:40ppb Co^{2+}:Cr^{6+}. Clusters of γ-H2AX positive cells (red foci) are ringed in white. The cell nuclei are counterstained with Hoechst-33342 (blue). The H9 cells were dual stained with Oct-4 (green) to eliminate contaminated MEFi cells or differentiated H9 cells from DNA damage score.

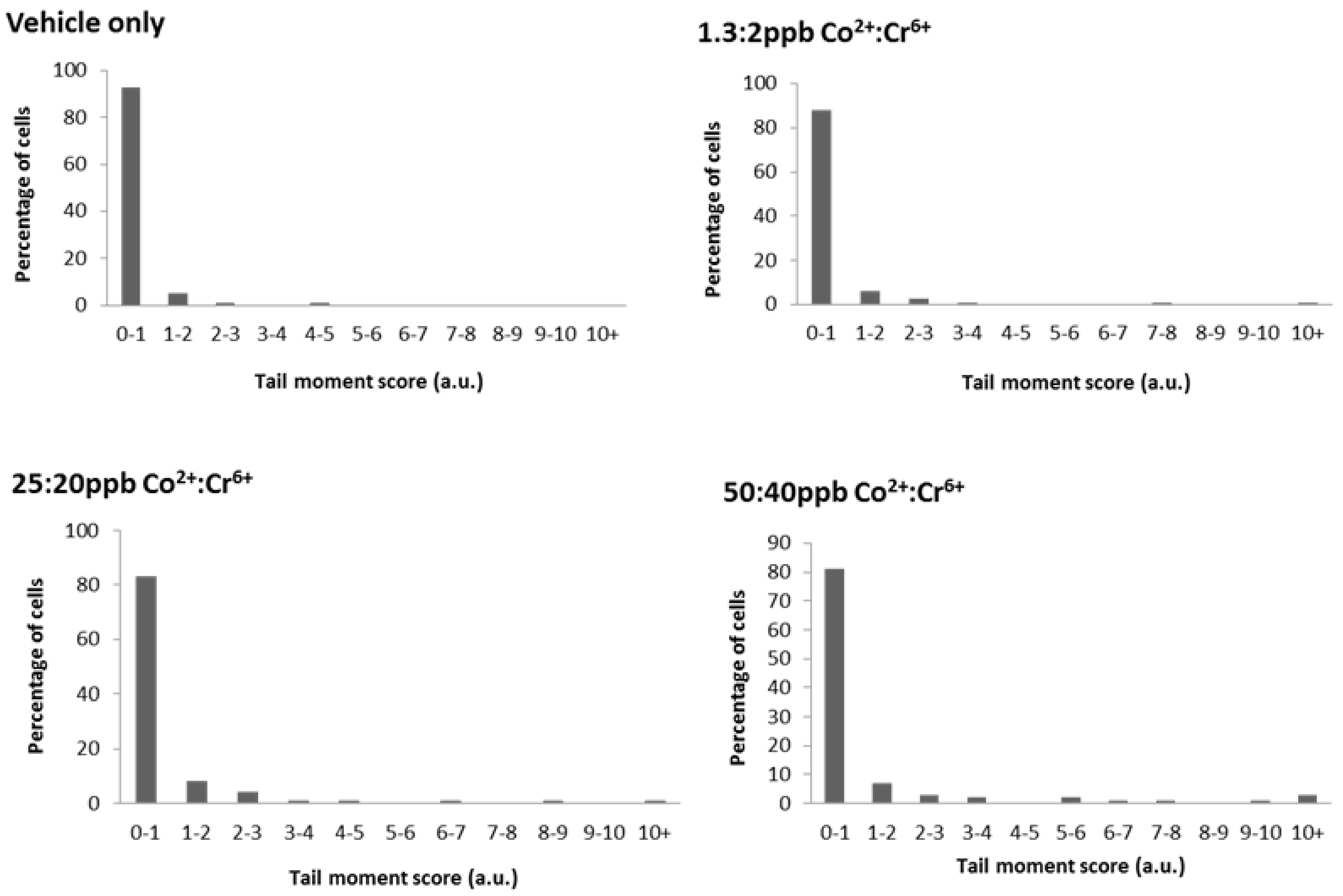

Suppl. Figure 5 Justification for the alkaline comet assay scoring system

hES cells with a tail moment greater than 3 were classified as DNA damaged because less than 1% of control hES cells have a tail moment greater than 3 compared to 3%, 5% and 10% after direct exposure to 1.3:2ppb, 25:20ppb and 50:40ppb Co^{2+}:Cr^{6+} (both known DNA damaging agents), respectively.

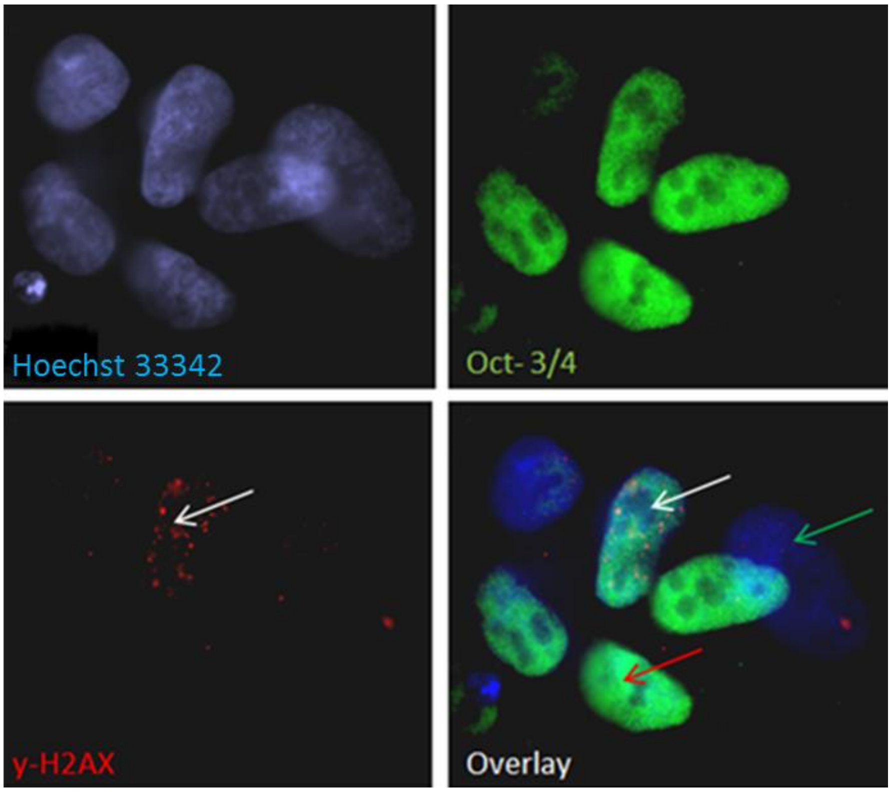

Suppl. Figure 6 Scoring the gamma H2AX assay

The image shows H9 cells stained with Hoechst-33342, Oct-4 and y-H2AX. Cell without Oct-4 staining (green arrow) were not included in the DNA damage scoring. The white arrow shows y-H2AX foci in an Oct-4 positive cell. The red arrow shows an Oct-4 positive cells with no y-H2AX foci.

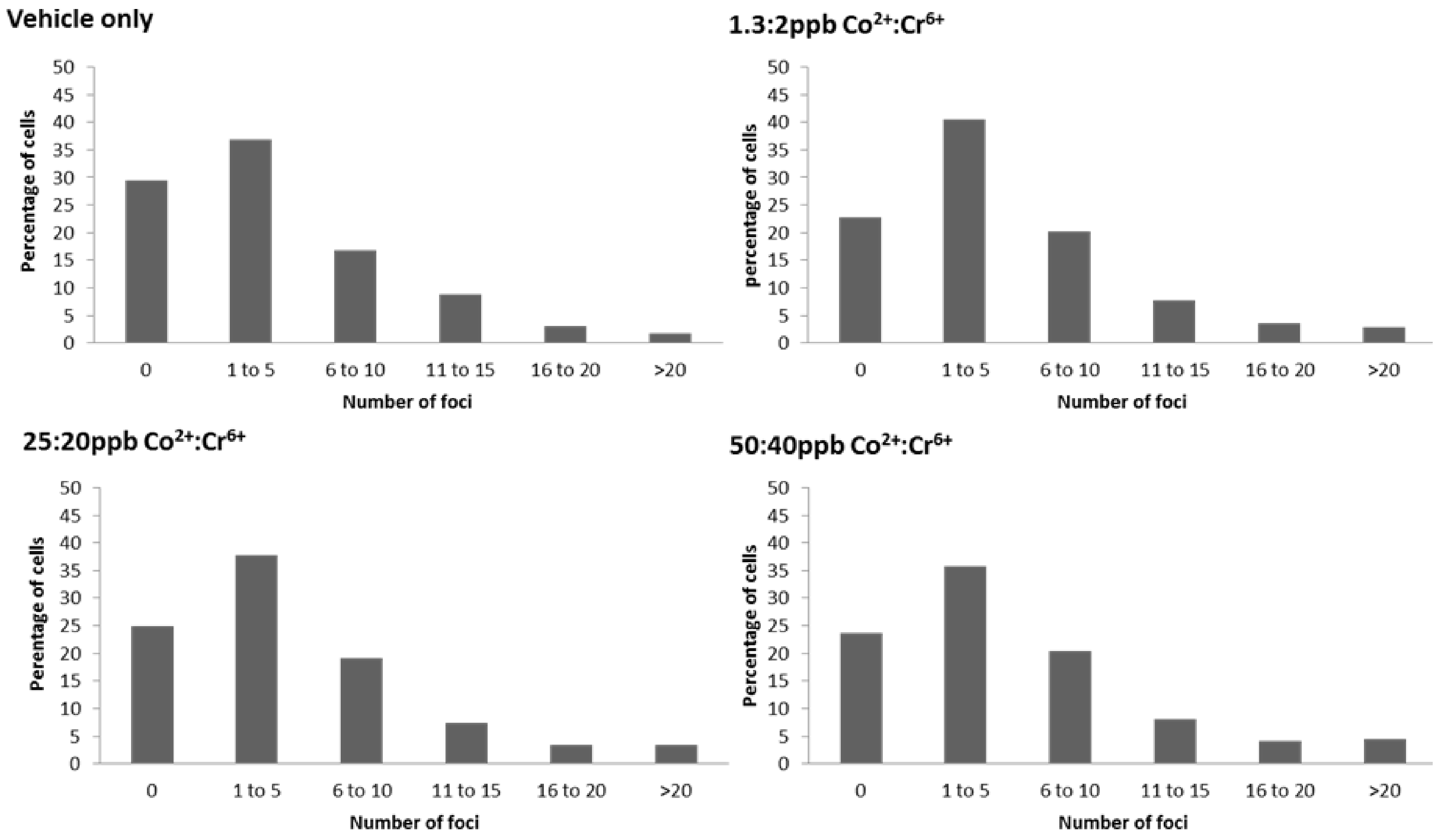

Suupl. Figure 7 Justification for the gamma H2AX assay scoring system

hES cells with more than 20 foci were classified as DNA damaged because less than 1.8% of control hES cells have more than 20 foci compared to 2.9%, 3.4% and 4.5% following direct exposure to 1.3:2ppb, 25:20ppb and 50:40ppb Co^{2+}:Cr^{6+} (both known DNA damaging agents) respectively.

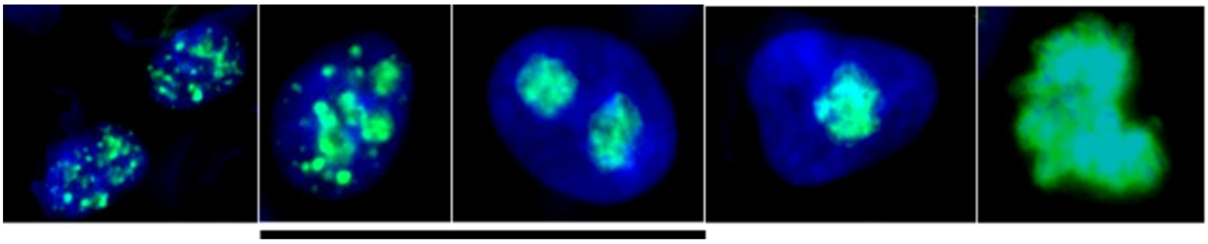

Suppl. Figure 8 Judgement of cell cycle phase from Ki67 nuclear staining patterns

The image shows H9 cell nuclei stained with Hoechst-33342 (blue) and Ki67 protein (green). Cells in early G1 have 30-50 small foci. Cells in late G1 and S have a smaller number of larger foci, gradually decreasing from 10 to 2. However, Ki67 staining alone cannot distinguish between G1 and S. Cells in G2 have just one large focus. M phase cells are recognised by their mitotic morphology and one large domain of Ki67 labelling. Ki67 staining is not present in G0 cells (not shown). Ki67 staining may also be seen in small foci in the cytoplasm, where Ki67 synthesis is taking place.

**List of primary antibodies**	**Associated secondary antibodies**
Phospho- Histone H2AX (Ser 139) (20E3) rabbit monoclonal antibody (Cell Signalling Technology #9718) diluted 1:400	Goat pAB to rabbit IgG (Dylight 550) (Abcam)
Cleaved Caspase-3 (Asp175) rabbit polyclonal antibody (Cell Signalling Technologies #9661) diluted 1:1600	As above
Monoclonal mouse anti-human Ki67 antigen clone M1B-1 (Dako #M724029-2) diluted 1:200	AlexaFluor 488 goat anti mouse IgG (Life Technologies)
Connexin 43 Monoclonal Antibody, Mouse (CX-1B1) (Invitrogen, Life Technologies #13-8300) diluted 1:200	As above
p53 (1C12) Mouse mAb antibody (Cell Signalling Technology #2524) diluted 1:2000	As above
Anti Oct- 3/4 (C-10) (Santo Cruz Biotechnology, Inc. #SC-5279) diluted 1:100	As above

Suppl. Table 1 List of primary and secondary antibodies
